# Sex- and gender-based medicine in pediatric nutrition

**DOI:** 10.1186/s13052-024-01734-6

**Published:** 2024-09-02

**Authors:** Veronica Maria Tagi, Giulia Fiore, Chiara Tricella, Francesca Eletti, Alessandro Visioli, Federica Bona, Gianvincenzo Zuccotti, Antonio Corsello, Elvira Verduci

**Affiliations:** 1 Department of Pediatrics, Vittore Buzzi Children’s Hospital, University of Milan, Milan, Italy; 2 Department of Biomedical and Clinical Science, University of Milan, Milan, Italy; 3 Department of Health Sciences, University of Milan, Milan, Italy; 4 Metabolic Diseases Unit, Department of Pediatrics, Vittore Buzzi Children’s Hospital, University of Milan, Milan, Italy

**Keywords:** Gender-based medicine, Sex-specific, Pediatric nutrition, Males, Females, Dietary reference values, Gender gap

## Abstract

Consistent evidence increasingly highlights the significance of integrating sex and gender medicine to ensure a precision approach according to individual patient needs. Gender discrepancies emerge across various areas, even from pediatric age. The importance of recognizing these differences in pediatric nutrition is critical for the development of targeted nutritional strategies and interventions, particularly in cases of associated pathologies, including obesity, metabolic-associated fatty liver disease, eating disorders, and inflammatory bowel disease. The review highlights the biological and sociocultural factors that contribute to different nutritional needs and health outcomes in male and female children. By examining current evidence, we underscore the necessity for precision medicine approaches in pediatric care that consider these sex- and gender-based differences. Moreover, differences in dietary requirements and dietary patterns between males and females are evident, underscoring the need for precise nutrition strategies for a more accurate management of children and adolescents. This approach is essential for improving clinical outcomes and promoting equitable healthcare practices. This review aims to provide an overview of nutrition-related medical conditions exhibiting sex- and gender-specific discrepancies, which might lead to distinct outcomes requiring unique management and prevention strategies. Future research and public health initiatives should address these differences in designing effective lifestyle education programs and nutrition interventions targeting both children and adolescents.

**Supplementary Information:**

The online version contains supplementary material available at 10.1186/s13052-024-01734-6.

## Introduction

According to the World Health Organization (WHO), gender refers to the socially constructed roles, behaviors, activities, and attributes that a society ascribes to men and women. Gender interacts but differs from sex, which refers to the different biological and physiological characteristics of females, males, and intersex persons. Gender identity, on the other hand, reflects the real feelings of the individual, internal sense of being male, female, or another gender, which may or may not align with their assigned sex at birth [1].

Gender Medicine represents a new approach to medicine aimed at recognizing and analyzing the differences arising from gender across various dimensions, including anatomy, physiology, biology, functionality, and social dynamics, as well as diverse responses to pharmacological treatments. This means that it does not simply deal with sex differences, but it focuses on a spectrum of different aspects [2]. Moreover, the role of pediatrics in supporting children in the development of their gender-related constructed role has not to be underestimated [3].

The significance of Gender Medicine gained recognition in 1991, when B. Healy published an article entitled “Yentl Syndrome”, which observed a sex bias in the management of coronary heart disease, resulting in poorer outcomes for women who underwent coronary angiography less frequently than men [4, 5].

Gender medicine is not new in pediatric and neonatal care. In 1971, Naeye et al. introduced the “hypothesis of the male disadvantage”, which describes the increased perinatal mortality in males compared with females [6]. In 2004, among the US population, the mortality rate for female infants was 6.08 per 1000, significantly lower than the same rate for males (7.44 per 1000), with a difference of 18% [7]. Moreover, a reduction of differences in health outcomes found among different population groups (e.g., gender, geographic area) represents a commitment for most national healthcare systems, even in high-income countries, since inequalities in infant/perinatal mortality rates persist [8]. In addition, Peacock et al. found that male sex in preterm newborns was significantly associated with increased rates of mortality, oxygen dependency, longer hospital stay, pulmonary hemorrhage, use of steroids, and major cranial ultrasound abnormalities [9].

Epidemiological data, as illustrated in Table 1, underscore disparities in the prevalence and characteristics of common pediatric issues between males and females. For instance, bronchiolitis exhibits a 50% greater incidence in males, who are more frequently hospitalized due to the development of severe infections [10, 11].

This review explores the role of Gender Medicine, identified as a broad concept referring to both genders, as well as the social construct that might affect the behavior of the community, the clinicians, and the patients, and the biological construct of sex, which may involve genetic, epigenetic and hormonal features influencing physiology and disease [12].

Specifically focusing on pediatric nutrition, our narrative review seeks to outline pathological conditions where sex- and gender-dependent nutritional disparities during childhood may lead to different health outcomes, necessitating tailored management and prevention strategies.

**Table 1:** Incidence rates of common pediatric conditions in males and females

	Males (%)	Females (%)	
Bronchiolitis [11]	1.5	1	*More severe in males*
Kawasaki Disease [13]	1.4–1.9	1	*Higher risk for coronary artery aneurysm in males*
Coeliac Disease [14, 15]	1	1.5–2	*Males and females develop different signs and symptoms*
Congenital Hip Dysplasia [16]	1	4	*Being female is a recognized risk factor*
Idiopathic Scoliosis [17]	1	1.4	*In females*,* it tends to progress more*
Precocious Puberty [18]	1	10	
Eating Disorders [19]	1	10	
Autism Spectrum Disorder [20]	4.4	1	*Underdiagnosed in females since symptoms may be milder*

## Methods

We conducted a narrative review to explore the sex- and gender-based differences in nutrition and nutrition-related diseases. We conducted extensive literature research on PubMed (Medline) and Scopus databases, including articles published from January 2000 to March 2024. Only English articles were included.

We aimed to study the sex- and gender differences in pediatric nutrition and several nutrition-related pathologies, namely obesity and metabolic syndrome, Metabolic dysfunction-associated steatotic liver disease (MASLD), eating disorders, coeliac disease (CD), anemia, and inflammatory bowel disease (IBD).

Starting from a total of 2747 papers, 2080 articles were excluded according to titles and abstracts. The authors then reviewed the full texts of the remaining papers and finally selected 114 relevant articles which were analyzed and included in the final review to provide a critical discussion. Additionally, the reference list of all articles was checked.

The list of keywords included in the research strategy, as well as the flowchart diagram of paper inclusion, are presented in Supplementary Materials (Table S1 and Figure S1).

## Sex- and gender-based medicine in nutrition

### Energy requirements

Nutrition naturally exhibits relevant sex-based differences, both in adult and pediatric ages. Females typically possess different body composition, energy requirements, and metabolism compared to males. Consequently, the nutrients required to maintain optimal health vary according to age and sex. Physical activity, dietary habits, and genetic background are also important determinants of nutritional status [21]. The term “dietary reference values” (DRVs) refers to a set of nutrient reference values that include the average requirement (AR), the population reference intake (PRI), the adequate intake (AI), and the reference intake (RI) for macronutrients. DRVs also include tolerable upper intake levels, which is the maximum amount of a nutrient that can be consumed safely over a long time [21]. Indeed, during developmental stages, energy requirements are influenced by both growth and nutrient requirements aimed at facilitating continuous increases in body mass. After the first year of life, the growth rate is relatively constant (5–6 g/day) until the onset of the pubertal phase, when it begins to increase (8 g/day at 8–9 years of age), reaching a new peak with puberty (about 13 g/day in females and 16 g/day in males), before declining thereafter [22, 23]. The primary factor influencing energy expenditure in the first two decades of life is the change in body mass: between 1 and 17 years of age, weight increases approximately six times in males and five times in females [23, 24]. Notably, lean mass increases during this phase, while the ratio of metabolically active organs to body weight decreases. Conversely, fat mass remains stable and comparable between genders until around ages 7–8, after which it gradually increases [23, 25]. The sex-dependent differences in body composition are accentuated with puberty, with boys experiencing greater increases in lean mass and girls in fat mass. Overall, the changes in body composition during the first two decades of life are complex, starting with puberty. In addition, the assessment of energy needs in the evolutionary age must consider the energy deposited in the tissues of neo-synthesis. The European Food Safety Authority (EFSA) [26], the Food and Agriculture Organization of the United Nations (FAO) [27] and the Scientific Advisory Committee on Nutrition (SACN) [28] agree to assume an energy cost for neo synthesis of approximately 2 kcal/g of weight increase for the period 1–17 years (Table 2).

**Table 2:** Energy requirements by sex and age according to the European Food Safety Authority (EFSA) [26]. Values reported are average requirements (AR) according to the lower level of physical activity (PAL) provided by the EFSA DRV. PAL is measured based on the ratio of total energy expenditure to basal metabolic rate. The parameters considered include activity type, intensity, and duration

	ENERGY KCAL/DAY		PAL
Years	Females	Males	
1	712	777	1.4
2	946	1028	1.4
3	1096	1174	1.4
4	1168	1256	1.4
5	1239	1332	1.4
6	1312	1409	1.4
7	1392	1497	1.4
8	1477	1592	1.4
9	1566	1684	1.4
10	1818	1933	1.6
11	1908	2043	1.6
12	2004	2174	1.6
13	2099	2333	1.6
14	2175	2513	1.6
15	2228	2699	1.6
16	2259	2845	1.6
17	2277	2940	1.6

### Dietary reference values

#### Macronutrients

According to EFSA DRVs for macronutrients, there are no sex-related differences in carbohydrate and lipid intakes during developmental age.

Nutritionally, two major categories of carbohydrates can be distinguished: “glycemic carbohydrates,” which are digested and absorbed in the small intestine, and dietary fiber, which are non-digestible carbohydrates reaching the large intestine [29, 30]. The absolute dietary requirement for glycemic carbohydrates is not well defined but depends on the amount of fat and protein ingested. EFSA proposes a value between 45 and 60 of total energy intake (E%) as the RI for carbohydrates, applicable to both females and males older than one year. Although a high frequency of intake of sugar-containing foods may increase the risk of dental caries, there is insufficient data to establish an upper limit for added sugar intake. Based on the available evidence on gut function, a fiber intake of 2 g/MJ is considered adequate for normal intestinal functionality after the first year of life [31, 32]. The EFSA Panel establishes an AI for dietary fibers at increasing values in the age range of 1–17 years, ranging from a minimum of 10 to a maximum of 21 g/day, independent of sex [33].

Fat intake during pediatric age can gradually be reduced from 40 E% at 6–12 months to 35–40 E% in toddlers (2–3 years). After 2 years of age, the proposed RI for fat intake ranges from 20 to 35 E%. Regarding the quality of fats, EFSA has provided specific recommendations regarding the intake of various fatty acids. For saturated fatty acids and trans fatty acids, it is recommended that their intake should be as low as possible [34]. Regarding cis-monounsaturated fatty acids, no DRV has been set. The available evidence does not support the establishment of a specific intake level for these fatty acids, which are found in foods such as olive oil and avocados. For total cis-polyunsaturated fatty acids (PUFA), the EFSA has also decided not to set a DRV. The decision is due to the lack of sufficient evidence to determine an optimal intake level. Cis-polyunsaturated fatty acids include omega-3 and omega-6 fatty acids, which are essential for various bodily functions. The EFSA has not set specific values for the ratio of n-3 to n-6 fatty acids. Although both types of fatty acids are important, the optimal ratio for health benefits is still uncertain, and therefore no specific recommendation can be made. An AI of 4% of total E% has been established for linolenic acid, a type of omega-6 fatty acid. This recommendation is aimed at ensuring that individuals consume enough linolenic acid to meet their nutritional needs. No DRV has been set for arachidonic acid, another type of omega-6 fatty acid. The evidence is not sufficient to establish a specific intake level for this fatty acid. The EFSA has also decided not to set an upper limit for total or any individual n-6 PUFA. There is no evidence indicating adverse effects from high intakes of these fatty acids, so no upper limit has been established. For alpha-linolenic acid (ALA), an omega-3 fatty acid, an AI of 0.5% of total E% has been set. This recommendation ensures that individuals consume enough ALA for health benefits. Additionally, no UL has been established for ALA, as there is no evidence of harm from high intakes of this fatty acid. Moreover, they suggest an AI of 250 mg for eicosapentaenoic acid (EPA) plus docosahexaenoic acid (DHA) from 2 years onwards, independently of sex, and an AI of 100 mg DHA for infants (> 6 months) and young children < 24 months [35–37].

Moreover, particularly in adolescence, females tend to gravitate towards healthier food choices, such as vegetarian-like diets, while males often prefer calorie-dense, fast foods, and red meat [38–40]. The protein requirements of children and adolescents are calculated by the factorial method considering what is needed for both growth and the maintenance of a progressively increasing protein mass. Concerning the specific needs for growth (synthesis and deposition of new tissues), the increase in protein mass of the body, and a protein utilization efficiency of 58% were considered [37]. For protein, AR and PRI values are the same for both sexes up to 10 years of age, thereafter from 11 years of age they tend to be higher in males. In absolute terms (g protein/day), since the age of 14, they differ markedly between boys and girls due to different body weights [41] (Table 3).

**Table 3:** Protein (g/kg/day); European Food Safety Authority (EFSA); PRI: Population Reference Intake

Age (years)	Protein (g/kg/day)	PRI
**12–17 months**	1.14	1.14
**18–23 months**	1.03	1.03
**2**	0.97	0.97
**3**	0.9	0.9
**4**	0.86	0.86
**5**	0.85	0.85
**6**	0.89	0.89
**7**	0.91	0.91
**8**	0.92	0.92
**9**	0.92	0.92
**10**	0.91	0.91
**11**	0.9	0.91
**12**	0.89	0.9
**13**	0.88	0.9
**14**	0.87	0.89
**15**	0.85	0.88
**16**	0.84	0.87
**17**	0.83	0.86

### Dietary patterns and eating habits

Several studies have shown the multifaceted influence of gender norms on dietary patterns, food trends, and perceptions of food and body image among children and adolescents. Regarding toddlers and preschool-aged children, no differences have been found in food trends [47, 48].

A cross-sectional study based on data from the Healthy Lifestyle in Europe by Nutrition in Adolescence Cross-Sectional Study (HELENA–CSS), identified three dietary patterns in boys (“snacking and bread”, “Mediterranean diet”, and “breakfast”) and four patterns in girls (“convenience”, “plant-based and eggs”, “Western”, and “breakfast”) [49]. A notable inverse correlation with increased adherence to overweight/obesity was only evident for the “breakfast” dietary pattern, observed in both girls and boys. The “breakfast” pattern identified in this study can be deemed the healthiest, independently of sex, due to its positive loading for fruit, milk, breakfast cereals, and dairy products, which are regarded as healthier food choices and integral components of a balanced diet. However, this pattern exhibited a negative loading for sugar-sweetened beverages in boys, and for cereals (such as pasta, rice, and others) in girls [49].

Evidence consistently shows gender-based differences in dietary patterns in middle childhood and adolescence, with females generally exhibiting more varied and healthier eating behaviors, such as higher consumption of fruits, vegetables, and plant-based foods, while males tend to consume more calorie-dense and fatty foods, including sugar and sweets, fast food and red meat [50–53]. Furthermore, higher consumption of full-fat milk, alcohol, and energy drinks has been described in males [54, 55]. As a result, this is reflected in the intake of specific nutrients, in facts sodium and potassium intake also exhibits gender-based differences. Boys tend to consume higher levels of sodium and potassium due to their preference for salty and high-potassium foods, while female intake could be influenced by overall dietary patterns and eventual health-conscious choices [56, 57].

Another study conducted on British students observed a higher tendency of girls to follow a “vegetarian” dietary pattern, while a “convenience, red meat & alcohol” pattern was preferred by boys [58].

Different hypotheses have been proposed to provide possible explanations for the observed disparities in food trends and dietary patterns. A neuroimaging study showed that boys aged 7 to 11 exhibited heightened activation in response to food compared to non-food images, particularly in the right posterior hippocampus and temporal occipital fusiform cortex—areas associated with memory and visual processing—in comparison to girls [59]. This finding contrasts with studies performed on adults, and therefore, the developmental course of neural reaction to food cues has yet to be fully elucidated [50].

Apart from variations in food-related neural processing, gender disparities may also stem from parental feeding methods, cultural emphasis on dieting, and peer influences. Indeed, gender norms and societal pressures significantly influence body image perceptions and dieting behaviors among adolescents. Females are more likely to avoid high-fat foods, consume fruits and fiber, and, to a lesser extent, restrict salt intake [60]. This is probably because they encounter greater food-related conflict compared to males, as they often enjoy high-calorie foods while simultaneously feeling they should not consume them [61]. In addition, girls more frequently experience body dissatisfaction, leading to a major engagement in dieting practices, which can lead to disordered eating patterns and negative health outcomes [62, 63]. Gender norms also play a role in shaping physical activity behaviors among girls. Societal expectations regarding femininity and body image may influence girls’ engagement in physical activity and their attitudes toward nutrition and weight control [64].

Gender differences in food choices therefore appear to be partly attributable to a greater weight control involvement among women, and partly to their stronger beliefs in healthy eating [60]. This may be also explained by the evidence that a higher intake of nutritious foods, such as fruits and vegetables, has an inverse correlation with depression [65]. Boys also seem to consume more fast foods due to a less accurate perception of what constitutes fast food [54, 66].

Overall, these findings underscore the importance of understanding the complex interplay between sex, gender norms, and dietary behaviors in adolescents.

## The impact on pediatric nutrition-related diseases

### Obesity and metabolic syndrome

The prevalence of obesity is steadily increasing globally [67], leading to an increase in obesity-associated complications such as diabetes mellitus, cardiovascular disease, hypertension, dyslipidemia, and liver disease [67, 68]. Pediatric obesity is among the priority goals on the healthcare agenda since the disease burden of obesity is well known to begin in early life [69].

A novel approach to reducing morbidity and mortality is to analyze sex- and gender-related characteristics in obese patients. Indeed, several differences between the male and female populations in adipose tissue distribution, endocrine, metabolic responses to nutritional interventions, and obesity complications have been recently observed [70–72].

According to multiple studies, adipose tissue (AT) distribution and type are essential in the development of obesity-related complications. AT is involved in a progressive process of growth and differentiation throughout life [73], beginning its deployment during the second trimester of gestation and lately representing endocrine most organ-secreting factors and hormones that will regulate metabolic homeostasis [71, 72, 74]. Although recent studies have shown different subtypes of AT, in humans, only the brown and white AT have been described [75, 76]. These two types of AT are both equally important for energy homeostasis but differ in distribution, lipid composition, and cytokine profiles [76]. The brown AT is associated with insulin sensitivity and increased energy expenditure [77].

Limited data are available regarding the assessment of adiposity before 5 years of age [78]. Fomon et al. described a low percentage of body fat (BF) growth rate between the ages of 2 and 5, having boys 19% and girls 20.4% body fat at 2 years [79]. Recently, Wells et al. provided reference data on adiposity from 6 weeks to 5 years old based on measurements of total body water (TBW) by isotope dilution [80], showing no significant differences between males and females. On the contrary, many studies have shown the sex differences in body fat composition during late childhood and adolescence. A study on Caucasian children observed that the percentage of BF was similar by sex until puberty. After this age, the percentage of BF decreased in boys but continued to gain progressively up to 18 years in girls [81]. A recent study in Southern Brazilians found a similar pattern of BF growth, with girls presenting higher adiposity with advancing ages. Although these studies have assessed body composition in children and adolescents of distinct ethnic origins, adiposity accrual follows a similar pattern across ethnicities.

Also, the pattern of adiposity distribution is sex-dependent [82], implying a different cardiovascular risk [83]. Indeed, since childhood females have more subcutaneous adipose tissue (SAT) with a “pear shape” (gynoid) distribution, while men have a predominantly greater amount of visceral adipose tissue (VAT) around the abdominal organs with an “apple shape” (android) body composition [71, 84]. This difference in adiposity distribution is accentuated in late puberty (Tanner stages 4–5), with boys having 17% greater trunk fat than girls [85]. Magnetic Resonance imaging data provided by Shen et al. confirmed these findings, showing a larger SAT volume in girls than in boys during puberty [86]. Such observations may be explained by the physiological predisposition in females to store energy, aiming for eventual pregnancies and lactation [87].

Greater visceral adiposity in men is correlated with elevated postprandial insulin, free fatty acids (FFA), and triglyceride levels, with an increased risk of developing metabolic complications [88, 89]. Indeed, the rate of lipolysis in VAT is higher than in SAT, causing an excessive FFA deposition in the liver and inducing gluconeogenesis and hyperinsulinism [90]. Moreover, some inflammatory modifications occurring in the VAT have been attributed to the development of metabolic complications [91].

Gender differences in body composition and adipose tissue are correlated with the effects of sex hormones [74]. Estrogen and progesterone receptors are mostly found on SAT [92]. In premenopausal females, the level of estrogen is higher with a protective effect against obesity complications [93]. It has also been observed that estrogen has a favorable effect on insulin sensitivity, as proven by the decrease in insulin sensitivity with menopause and subsequent improvement with estrogen replacement [92]. Moran et al. demonstrated that, while in males insulin resistance (IR) worsens during puberty despite a decrease in adiposity, in females there are no important changes in IR, despite an increase in body fat [94].

The adipokines leptin and adiponectin may also have a role in sex differences [95]. Leptin activates the hypothalamus to secrete a gonadotropin-releasing hormone that activates the pituitary gland to produce follicle-stimulating hormone and luteinizing hormone, resulting in the onset of menarche [95].

Leptin concentrations appear to be four times higher in females than in males [92, 96]. The reasons for this finding are still unclear [97]. However, it has been observed that androgens have a negative association with leptin concentrations in men [98], while estrogens induce an increase in leptin concentrations [92, 99].

Adiponectin is a hormone secreted by the AT [100]. It lowers glucose production in the liver and improves insulin sensitivity in both muscles and the liver by increasing FFA oxidation [100]. Adiponectin levels have been reported to be significantly higher in women than in men, even after adjusting for differences in BMI [101].

Some research conducted on adults also showed that resting energy expenditure (REE), measured by indirect calorimetry, is significantly lower in women than in men [102]. According to other studies, sex does not appear to play a role since the REE is similar when normalized to kilograms of body weight or kilograms of lean body mass, the major contributor to REE [92]. Even ghrelin secretion may be sexually dimorphic. Some studies, despite conflicting results, report differences in concentration between males and females, who seem to reach higher levels of ghrelin [103, 104].

There might be a sex-dependent endocrine response to nutritional intervention. According to the European Union Childhood Obesity Project, the IGF1 axis responses to high protein (HP) formulas were modulated by sex, showing that total and free IGF-1 and IGF binding protein 3 concentrations were higher in girls than in boys [72, 105]. A study conducted by Closa-Monasterolo et al. investigated whether infant feeding choices can modulate later obesity and obesity complications [72]. According to these studies, a higher early protein intake is associated with higher IGF-1 and lower IGF-binding protein 2 secretion, and apparently, this response is stronger in girls than in boys [72]. It was also shown that the leptin concentration was higher in females than in males. The IGF-1 axis of female infants shows a stronger response to the nutritional intervention than does that of male infants [72].

New indexes of adiposity have been introduced to assess the correlation between body-fat distribution and cardiometabolic risk. However, data on the correlation between IR and these new indexes are limited. Some studies evaluated the relationship between IR and adiposity indexes in children and adolescents with obesity, focusing on gender differences. Calcaterra et al. investigated a cohort of 586 patients with obesity [106]. The Triglyceride-Glucose (TyG) index is a surrogate marker of insulin resistance calculated from fasting triglyceride and glucose levels, while the Homeostatic Model Assessment of Beta-cell Function (HOMA-β) is a method used to quantify insulin secretion [106–108]. BMI significantly correlated with all IR parameters except for the TyG index in females. Fat mass was associated with IR parameters only in females; BMI-z score with IR markers except for HOMA-β in males; waist-to-height ratio with HOMA-β in both sexes [106]. Triglycerides were correlated with all IR indexes in both sexes [106]. Correlations between different sex parameters were significantly more evident in middle puberty. The relationship between IR surrogate markers and obesity indexes is influenced by gender in pediatrics [106]. Sex-specific differences in obesity-related complications should be considered in preventive intervention decision-making.

These data suggest that adiposity at birth and during childhood is correlated with the risk of developing obesity and obesity complications later in life. Therefore, there is an important window of opportunity for prevention and decreasing the predisposition toward obesity. Moreover, the sex and gender differences in adipose tissue and obesity condition should regarded as a new way to strategize treatment and prevention measures.

### Micronutrients

For the general population, international recommendations are available under the form of RDA (recommended dietary allowances), or more recently, as DRV [33, 42]. Zinc values AR are the same for both sexes until the age of 14. Thereafter, the values change increasing more for males than for females. This increase is due to seminal fluid losses in male adolescents [43]. On the other hand, iron DRV has the same values until the age of 10, after which the value for females increases due to menstrual cycle losses. Regarding fluoride, even though no AR for the performance of essential physiological functions can be defined, the EFSA Panel considered that the setting of an AI is appropriate because of the beneficial effects of dietary fluoride on the prevention of dental caries [44]. Fluoride AI is different according to sex, being higher in males. Conversely, selenium, chloride, and calcium values are superimposable for both sexes from 1 to 17 years (Table 4).

**Table 4:** Micronutrient – minerals DRV from the european food safety authority (EFSA)

Micronutrients - Minerals										
	**1–3 YEARS**		**4–6 YEARS**		**7–10 YEARS**		**11–14 YEARS**		**15–17 YEARS**	
	**F**	**M**	**F**	**M**	**F**	**M**	**F**	**M**	**F**	**M**
**ZINC (mg/day) PRI**	4.3	4.3	5.5	5.5	7.4	7.4	10.7	10.7	11.9	14.2
**SELENIUM (µg/day) AI**	15	15	20	20	35	35	55	55	70	70
**FLUORIDE (mg/day) AI**	0.6	0.6	0.9	1	1.4	1.5	2.3	2.2	2.8	3.2
	**1–3 YEARS**		**4–6 YEARS**		**7–10 YEARS**		**11–17 YEARS**			
	**F**	**M**	**F**	**M**	**F**	**M**	**F**	**M**		
**CHLORIDE (g/day)** *Safe and adequate intake*	1.7	1.7	2	2	2.6	2.6	3.1	3.1		
	**1–3 YEARS**		**4–10 YEARS**		**11–17 YEARS**					
	**F**	**M**	**F**	**M**	**F**	**M**				
**CALCIUM (mg/day) PRI**	450	450	800	800	1150	1150				
**IRON (mg/day) PRI**	7	7	11	11	13	11				

Abbreviations: Population Reference Intake (PRI); Adequate Intake (AI)

**Table 5:** Micronutrient - vitamins from the European Food Safety Authority (EFSA). RAE: retinol equivalents; PRI: Population Reference Intake; AI: adequate intake; α–TE: alpha-tocopherol

Micronutrients - Vitamin								
	**Vit. A (µg/RE day) PRI**		**Vit. C (mg/day) PRI**		**Thiamine (µg/day) PRI**		**Riboflavin (µg/day) PRI**	
	**F**	**M**	**F**	**M**	**F**	**M**	**F**	**M**
**1–3 YEARS**	250	250	20	20	0.1	0.1	0.6	0.6
**4–6 YEARS**	300	300	30	30	0.1	0.1	0.7	0.7
**7–10 YEARS**	400	400	45	45	0.1	0.1	1	1
**11–14 YEARS**	600	600	70	70	0.1	0.1	1.4	1.4
**15–17 YEARS**	650	750	90	100	0.1	0.1	1.6	1.6
	**Vit. E / α–TE (mg/day) AI**							
	**F**	**M**						
**1–2 YEARS**	6	6						
**3 YEARS**	9	9						
**4–6 YEARS**	9	9						
**7–9 YEARS**	9	9						
**10 YEARS**	11	13						
**11–14 YEARS**	11	13						
**15–17 YEARS**	11	13						

Regarding vitamins, the available data are superimposable for both sexes, the only differences are noted in vitamin E, vitamin A, and vitamin C (Table 5). For Vitamin A and C differences in the PRI arise in the 15–17 age group, where a significant increase emerges for males. Since Vitamin A comprises a group of fat-soluble molecules (preformed vitamin A and provitamin), that possess the biological activity of retinol, the total amount of Vitamin A is expressed as retinol equivalents. Regarding vitamin C, in accordance with the EFSA document [45], it is recommended not to exceed the tolerable upper intake level, equivalent to 1 g/day. Lastly, for vitamin E, sex-difference AI is provided from 10 years onwards. Because of the presence of different forms of Vitamin E, in nutritional terms, the amounts of vitamin E are expressed as α tocopherol equivalents (α - TE) [45, 46].

**Fig. 1:**
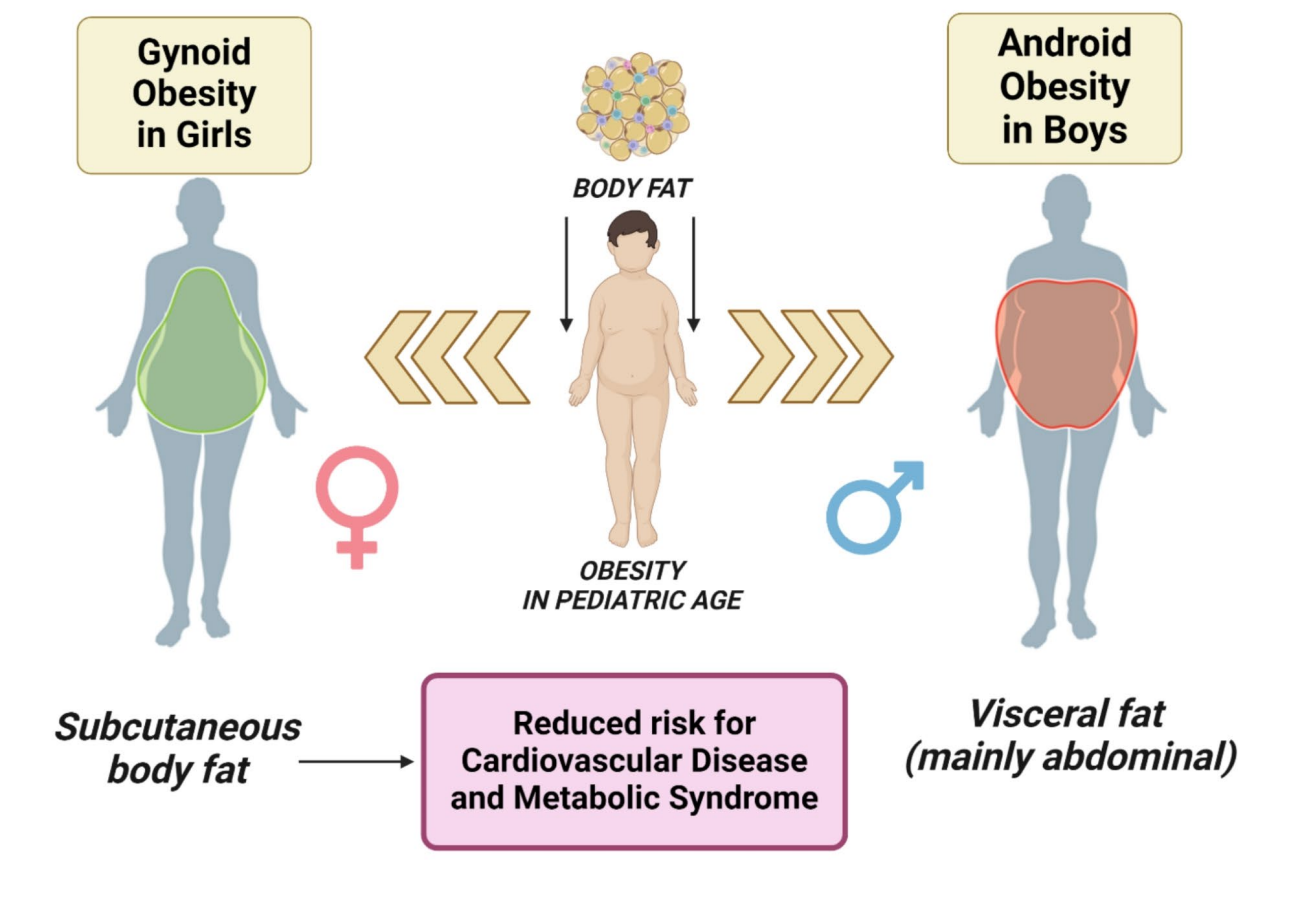
Sex-related body fat distribution

Figure 1 gives an overview of sex-based differences in body composition.

### MASLD

Non-alcoholic fatty liver disease (NAFLD) is a chronic liver disease characterized by fat accumulation in the liver [109, 110]. To better describe the differences between patient presentations, pathogenesis, and management, the terminology has been updated to MASLD, to underly its association with metabolic dysfunction [111].

Pediatric MASLD is a chronic hepatic steatosis, the pathogenesis of which is not linked to genetic/metabolic disorders, infections, medication use, ethanol consumption, or malnutrition. In most cases, MASLD in children is linked to insulin resistance, obesity, and dyslipidemia, which is characterized by high triglyceride and low high-density lipoprotein cholesterol (HDL-C) levels [109]. Histologically, NAFLD is divided into the non-alcoholic fatty liver (NAFL), characterized by mild steatosis, and non-alcoholic steatohepatitis (NASH), defined by steatosis, lobular inflammation, and hepatocellular injury [109]. Nowadays, MASLD is the most important form of chronic liver disease worldwide, which is linked to the rising rates of obesity [112–114]. It is reported that the global prevalence of MASLD is approximately 34% among overweight or obese children aged 1–19 years, and 45% in those attending child obesity clinics [115].

Evidence suggests that MASLD is more common in males than females. A Mexican study [116] involving 194 participants found that MASLD is more frequent in boys. Authors reported that only alanine aminotransferase (ALT), and no other clinical or metabolic values, were associated with MASLD in boys. In contrast, Homeostatic Model Assessment for Insulin Resistance (HOMA-IR), Visceral Adiposity Index (VAI), triglyceride levels, and ALT were associated with MASLD only in girls [116].

Ayonrinde et al. [117] conducted a study on 1170 Australian adolescents. The prevalence of MASLD was 12.8%. Females had a significantly higher prevalence of MASLD (16.3% versus 10.1%), and central obesity (33.2% versus 9.9%) compared to males. The severity of hepatic steatosis was linked with several variables, such as body mass index, waist circumference, subcutaneous adipose tissue thickness (SAT), serum leptin level, HOMA-IR, and serum alanine aminotransferase level in both sexes. However, the association between visceral adipose tissue thickness and decreased serum adiponectin levels was found only in males [117]. Despite the lower prevalence of MASLD in males, their phenotype was associated with a higher level of adverse metabolic events and greater visceral adiposity compared to females.

Further research is needed to better understand gender and sex differences in children with MASLD. Many studies on adults show sex-specific variations in MASLD, with protective effects linked to female sex hormones. Women generally have a lower risk of MASLD during reproductive age, with this protective effect diminishing after menopause. The use of hormone replacement therapy (HRT) in postmenopausal women seems to lower the risk of MASLD [118, 119].

These findings describe the importance of MASLD screening, particularly in males. Further research is needed to better understand sex-related mechanisms of MASLD, especially in children and adolescents.

### Eating disorders

Eating disorders (EDs) are psychiatric pathological conditions commonly observed during early to late adolescence, a critical period for neural, physical, and psychological development. While the pathogenesis of EDs remains elusive, an apprehensive approach to weight, body shape, and eating behaviors plays a pivotal role in their onset. If untreated, EDs can lead to significant acute and long-term consequences [120]. Six main eating disorders are recognized in diagnostic systems: anorexia nervosa, bulimia nervosa, binge eating disorder, avoidant-restrictive food intake disorder, pica, and rumination disorder [121]. EDs severely impact both males and females health [120, 122]. Nowadays the age of onset has dropped and there has been an increase in “non-specific” forms in both genders [122]. While traditionally considered more frequent in females, recent trends indicate an increased prevalence of EDs in males. The male-female ratio for anorexia and bulimia has changed from one affected male for every ten females to one male for every four females affected by anorexia, and one male for every 8–11 females affected by bulimia [121]. Girls still have a higher risk of developing anorexia nervosa or bulimia nervosa. Conversely, gender differences seem to be less pronounced for binge EDs. EDs impact both genders, with varying presentations: men may focus on muscularity, while women usually emphasize weight loss [121, 123]. The majority of the reports on EDs show poor gender-specific results, especially because of small samples or the exclusion of males [123].

Recent studies describe distinct subgroups among those at risk for EDs, including models, dancers, and athletes. Individuals who practice certain sports, such as bodybuilders, wrestlers, swimmers, runners, rowers, gymnasts, and jockeys, have a higher risk of developing eating disorders, related to the weight restrictions typical of these sports. Body dissatisfaction and dieting are risk factors for EDs [124]. Body dissatisfaction is especially linked to an increased risk of eating disorders in adolescent females [125].

Støving et al. evaluated gender differences in weight restoration in various EDs in 1015 patients with anorexia nervosa, eating disorders not otherwise specified, and bulimia nervosa [123]. The authors found that remission rates were lower in females, suggesting a better outcome for males. Marques et al. described a gender-specific pattern in EDs. Females have a higher rate of cognitive symptoms associated with EDs, while both genders endorsed eating restraint equally [126].

### Coeliac disease

The CD is a chronic immune-mediated enteropathy triggered by the intake of dietary gluten and related proteins in genetically susceptible individuals [127]. It has become one of the most common food-related chronic intestinal diseases among children, with an estimated prevalence of 1-1.4% of the population worldwide [127]. Besides genetic and immunological factors, it appears that other environmental determinants, including dietary patterns, can play a significant role in the development of CD [128].

During childhood, CD seems to show some peculiar sex-related clinical differences [110]. For instance, in Western countries, there is a female predominance of the CD [128]. This gender predominance is found in both children and adults.

CD can appear with a wide spectrum of clinical manifestations [110]. In recent years, there has been a shift in the clinical presentation of CD from the classical form to the non-classical, oligosymptomatic, and asymptomatic forms [128]. The classical form of CD is more frequent in females, with symptoms like anemia and abdominal bloating more commonly observed. [128]. Non-classical presentations are more prevalent in males, who also have a higher percentage of silent manifestations and later diagnosis [15, 129]. Moreover, females have a significantly higher risk of developing lymphoma, highlighting a further gender disparity [128].

A study by Megiorni et al. found that in DQ2/DQ8 negative patients there is an unexpected male predominance, showing a possible role of HLA genes on the sex-related different clinical presentations and suggesting a possible epigenetic effect on the two sexes [130].

Moreover, Jansson-Knodell et al. observed a higher risk of CD in women than in men in the undiagnosed populations [118], underlying a clear gender disparity in undiagnosed CD [14]. Screening studies have identified a higher number of girls compared with boys, and of adult women compared with men [14].

Therefore, considering the increased risk for CD among females and gender differences is crucial for screening, diagnosis, and management strategies.

### IBD

IBDs, including ulcerative colitis (UC) and Crohn’s disease (CrD), are commonly seen in pediatric age, as their onset often occurs during adolescence and young adulthood. Approximately 25% of patients with IBDs manifest before the age of 20 [131].

Although immune-mediated diseases typically show a female preponderance, this does not always appear to be true for IBDs: in Europe and the United States, CrD prevalence appears to be higher in females, while in Asia the opposite has been observed. Young females aged 10–14 years show a 20% lower risk for CrD in comparison with males. In contrast, 25 to 29-year-old girls or women older than 35 years seem to be more prone to be diagnosed with CrD compared to their male counterparts [132]. Regarding environmental risk factors, it has been reported that smoking and appendicectomy seem to be more determined in females than in males. On the other hand, the use of antibiotics tends to be a more important determinant in male patients.

Gender-specific differences in IBD have been reported in clinical presentation, disease course, complications, response to therapies, adherence, psychosocial functioning, and psychiatric co-morbidities [132, 133].

As far as genetic predisposition is concerned, female preponderance appears to be higher in familial cases compared to sporadic IBD cases (61 vs. 54%). Genetic studies have identified more than 230 loci associated with IBDs. Some gene variants, such as IL-23R, protect females but not males. Conversely, other variants, like a single nucleotide polymorphism in the promoter region of IL-10, offer protection to males [132, 133].

Disease phenotype may differ in men and women: females develop extraintestinal manifestations more often than men. On the other hand, males with CrD are more prone to show involvement of the upper gastrointestinal tract and ileal disease. In addition, IBD-related complications show an association with sex: colorectal cancer arising in IBDs appears to be higher in men than in women, and pulmonary complications show higher mortality in female patients affected by CrD. Moreover, osteopenia and osteoporosis were more frequently reported in male than female IBD patients [133].

Responses to IBD treatment also seem to vary between boys and girls: male sex has been shown to correlate with loss of response to Tumor Necrosis Factor blockers and more often require dose intensifications. Females develop more adverse effects more frequently during TNF blocker administration, thus reducing adherence. Neglecting these gender-specific aspects may result in suboptimal treatment [132].

The prevalence of depression is higher among female patients. One of the most disabling symptoms seems to be fatigue, which has a higher prevalence among women of all age groups in comparison to men. Finally, the impact of IBDs on body image, particularly in females, should be taken into account while considering gender differences in disease acceptance [132, 134, 135].

### Anemia

Anemia is defined by hemoglobin levels two standard deviations below the mean for age. The growing rates of restrictive diets in Western countries, often due to ideological reasons, are leading to macro- and micronutrient deficiencies. These deficiencies can cause even significant diseases with potentially irreversible damage to organs like the CNS, especially if they occur in prenatal life, due to possible epigenetic effects of nutrition in intrauterine life and developmental age [136, 137]. Age-based mean hemoglobin levels are shown in Table 6. After the age of 12, hemoglobin normal levels can be further divided into gender-specific ranges (Table 6) [138, 139].

**Table 6:** Hemoglobin levels in children and adolescents, after 12 years of age values are both sex- and age-specific. Data extracted from Wang et al. [138]

Age	Mean hemoglobin level	-2 Standard Deviation
Birth (term infant)	16.5 g/dL (165 g/L)	13.5 g/dL (135 g/L)
1 month	13.9 g/dL (139 g/L)	10.7 g/dL (107 g/L)
2 months	11.2 g/dL (112 g/L)	9.4 g/dL (94 g/L)
3–6 months	11.5 g/dL (115 g/L)	9.5 g/dL (95 g/L)
6 months – 2 years	12 g/dL (120 g/L)	10.5 g/dL (105 g/L)
2–6 years	12.5 g/dL (125 g/L)	11.5 g/dL (115 g/L)
6–12 years	13.5 g/dL (135 g/L)	11.5 g/dL (115 g/L)
12–18 years		
*- Males*	14.5 g/dL (145 g/L)	13 g/dL (130 g/L)
*- Females*	14 g/dL (140 g/L)	12 g/dL (120 g/L)

Anemia is a prevalent condition affecting approximately 40% of children globally, with potential long-term complications such as failure to thrive and neurological development impairments. [138]. It may be caused by nutritional deficiencies (most commonly iron deficiency), chronic illnesses, genetic conditions (such as thalassemia or sickle cell anemia), blood loss, and hemolysis [138, 139].

As far as sex-specific features are concerned, it is well known that iron deficiency is more common among premenopausal women due to menstrual blood loss [140], but it is also noteworthy to illustrate that a sex-based difference in vitamin B12 levels may exist: Margalit et al. observed that men are more susceptible to vitamin B12 deficiency, leading to macrocytic anemia. This does not seem related to different diet habits or hormonal effects. Therefore genetic variations between males and females may have an influence [141]. It has to be further noted that the expression of genetic diseases may even vary depending on the mode of inheritance. For instance, X-linked diseases often present differently in males and females due to the presence of one or two X chromosomes, creating further gaps and paradigmatic considerations [142, 143].

Additionally, gender-related social habits may play a role. In India, 50% of women of reproductive age, compared with 23% of men, have iron deficiency anemia, partly associated with a commonly adopted vegetarian diet. However, a study was conducted to investigate if gender-associated norms in this geographical area may influence the prevalence of anemia [144]. Although some of the reasons for higher rates of anemia in women compared with men are biological (iron loss during menstruation and sharing nutrients during pregnancy), social determinants were observed to study these disparities as well: due to a double burden of work, women would lack the time to visit a health center to get tested or obtain supplements. Women were also expected to prioritize the health of other family members over theirs. This should be kept in mind while treating patients from different cultural backgrounds [144].

## Conclusions

The influence of sex and gender on human health and disease is an emerging subject of study but is still underestimated and understudied in medical practice.

Understanding the diverse nutritional requirements and dietary habits according to gender is crucial for guiding clinicians in tailoring nutritional interventions for pediatric patients. Dietary considerations should be gender-specific from an early age and incorporated into the health management of children and adolescents. Dietary habits and adherence to dietary patterns are strongly influenced by environment, social norms, and ethnicity, particularly in adolescence. Recent studies have shown gender roles in the establishment of dietary patterns and eating habits. Addressing gender-specific factors and societal influences is crucial in designing effective nutrition education programs and interventions targeting adolescents. Promoting healthier dietary patterns and body image perceptions requires a comprehensive approach that considers individual, familial, societal, and environmental factors.

In conclusion, several nutrition-related diseases, including obesity, MASLD, EDs, anemia, CD, and IBD, present sex and gender-related features starting from the pediatric age. Therefore, understanding these differences in nutrition and nutrition-related pathological conditions is fundamental for the correct management of affected children and adolescents.

Efforts to integrate sex and gender considerations into medical research, practice, and education are urgently needed to ensure gender parity in healthcare. Targeted therapeutic strategies, particularly precision nutritional interventions in pediatric age groups, are becoming increasingly important.

### Electronic supplementary material

Below is the link to the electronic supplementary material.

Supplementary Material 1: **Table S1.** List of keywords used for literature research for each outcome of interest.

Supplementary Material 2: **Fig. S1**. Flowchart process of article selection

## Data Availability

Not applicable.
