# Supplementary material for: Sex- and gender-based medicine in pediatric nutrition
Source: Ital J Pediatr. 2024 Sep 2;50:159. doi: 10.1186/s13052-024-01734-6 (PMC11368030; doi:10.1186/s13052-024-01734-6)

**SUPPLEMENTARY MATERIALS**

**Table S1.** List of keywords used for literature research for each outcome of interest.

| **Outcome** | **Keywords** |
| --- | --- |
| Dietary reference values | NA (EFSA, FAO and SACN guidelines were adopted) |
| Dietary patterns and food trends | “DIETAY PATTERN” OR “FOOD TREND” AND “GENDER” OR “SEX” AND “CHILDREN” OR “CHILDHOOD” OR “ADOLESCENT” OR “ADOLESCENTS” OR “PEDIATRIC” |
| Obesity and Metabolic Syndrome | “OBESITY” OR “METABOLIC SYNDROME” AND “GENDER” OR “SEX” AND “CHILDREN” OR “CHILDHOOD” OR “ADOLESCENT” OR “ADOLESCENTS” OR “PEDIATRIC” |
| MASLD | “METABOLIC ASSOCIATED FATTY LIVER DISEASE” OR “MAFLD” OR “MASLD” AND “GENDER” OR “SEX” AND “CHILDREN” OR “CHILDHOOD” OR “ADOLESCENT” OR “ADOLESCENTS” OR “PEDIATRIC” |
| Eating disorders | “EATING DISORDER” OR “ANOREXIA” OR “BULIMIA” OR “BINGE EATING” OR “AVOIDANT-RESTRICTIVE FOOD INTAKE” OR “PICA” OR “RUMINATION” AND “GENDER” OR “SEX” AND “CHILDREN” OR “CHILDHOOD” OR “ADOLESCENT” OR “ADOLESCENTS” OR “PEDIATRIC” |
| Coeliac disease | “COELIAC DISEASE” AND “GENDER” OR “SEX” AND “CHILDREN” OR “CHILDHOOD” OR “ADOLESCENT” OR “ADOLESCENTS” OR “PEDIATRIC” |
| Anemia | “ANEMIA” OR “HEMOGLOBIN” AND “GENDER” OR “SEX” AND “CHILDREN” OR “CHILDHOOD” OR “ADOLESCENT” OR “ADOLESCENTS” OR “PEDIATRIC” |
| IBD | “INFLAMMATORY BOWEL DISEASE” OR “IBD” OR “CROHN” OR “ULCERATIVE COLITIS” AND “GENDER” OR “SEX” AND “CHILDREN” OR “CHILDHOOD” OR “ADOLESCENT” OR “ADOLESCENTS” OR “PEDIATRIC” |

Abbreviations: not available (NA); European Food Safety Authority (EFSA); Food and Agriculture Organization of the United Nations (FAO); Scientific Advisory Committee on Nutrition (SACN), Metabolic dysfunction-associated steatotic liver disease (MASLD); Inflammatory Bowel Disease (IBD)

**Figure S1.** Flowchart process of articles selection.


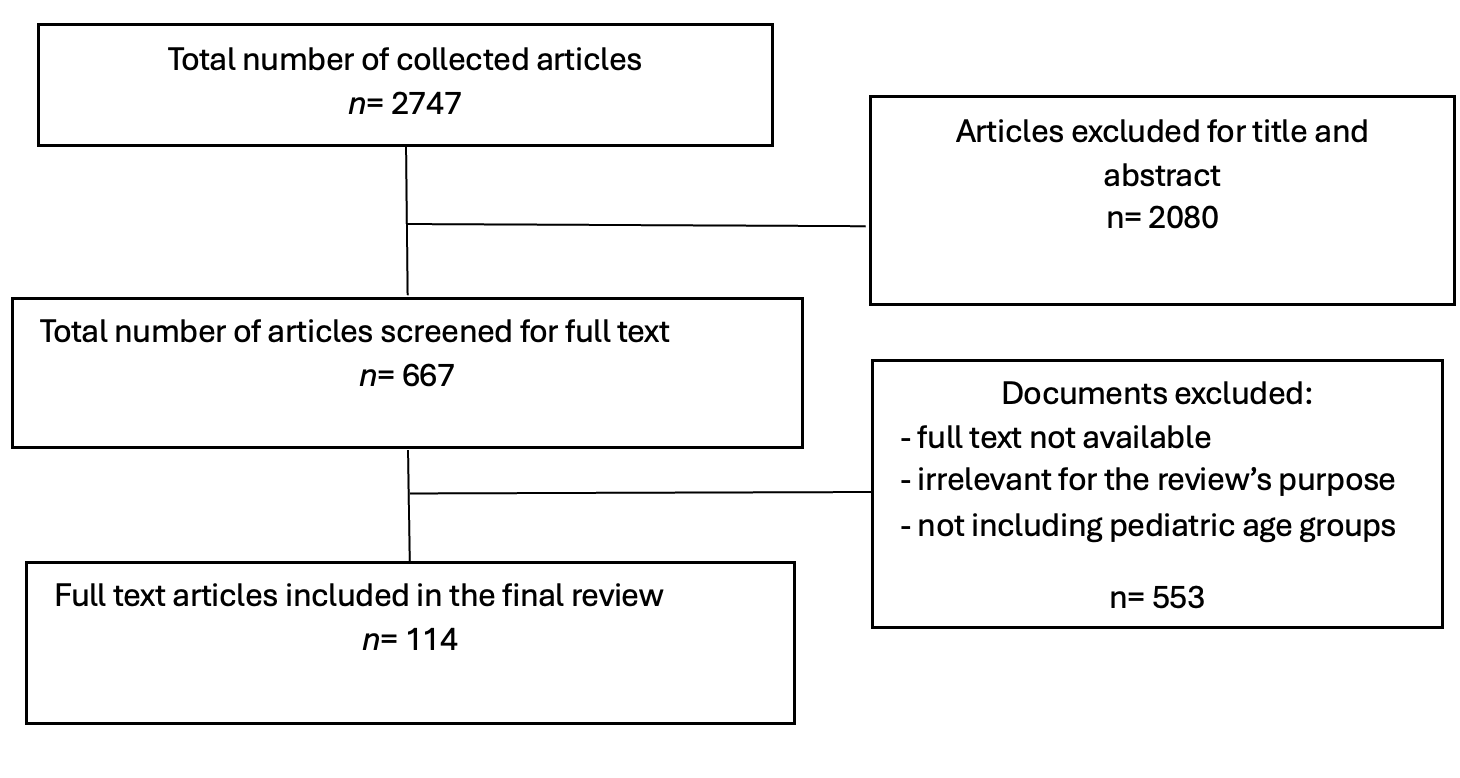

Supplement: Supplementary file 1 — Supplementary Material 1: Table S1. List of keywords used for literature research for each outcome of interest. [file 13052_2024_1734_MOESM1_ESM.docx]
