# Supplementary figures and images for: Sex- and gender-based medicine in pediatric nutrition
Source: Ital J Pediatr. 2024 Sep 2;50:159. doi: 10.1186/s13052-024-01734-6 (PMC11368030; doi:10.1186/s13052-024-01734-6)

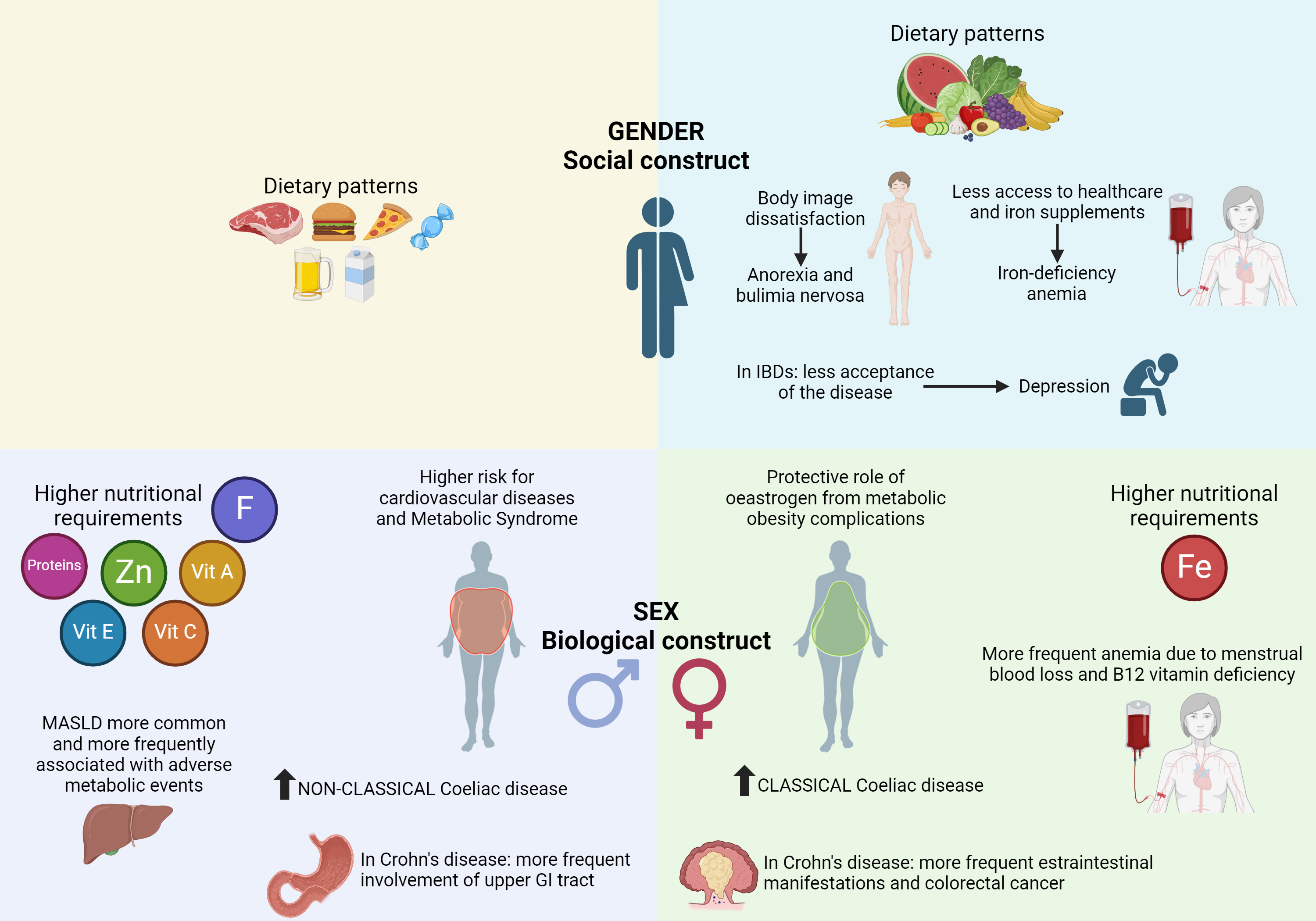

Supplement: Supplementary file 2 — Supplementary Material 2: Fig. S1. Flowchart process of article selection [file 13052_2024_1734_MOESM2_ESM.png]
